# Draft Genome Sequence of a Humic Substance-Degrading *Paenibacillus* sp. Isolated from the Subarctic Grasslands at Low Temperature

**DOI:** 10.1128/genomeA.00170-12

**Published:** 2013-02-14

**Authors:** Ha Ju Park, Dockyu Kim

**Affiliations:** Division of Life Sciences, Korea Polar Research Institute, Incheon, Korea

## Abstract

The *Paenibacillus* sp. strain PAMC 26794 was isolated from the tundra grasslands in Alaska for its high ability to degrade humic acids. We sequenced the PAMC 26794 genome to discover the degradative genes for natural humic substances and we propose the degradation pathway(s) of an abundant bacterial group (genus *Paenibacillus*) that inhabits cold environments.

## GENOME ANNOUNCEMENT

Humic substances (HSs), an important fraction of soil organic carbons, are distributed widely in low-temperature environments, including the Arctic and the Antarctic (1–3). Amorphous and high-molecular-weight HSs are thought to be composed of aromatic, aliphatic, phenolic, quinonic, and N-derived components, which are bonded covalently. HSs are synthesized via the decomposition of plant material and other organic matter, and then through the condensation of smaller molecules through biological and physical processes (2). Until now, much research has been centered on the structure, distribution, and reaction chemistry of HSs as a new source of fine chemicals and bioenergy (4). However, in spite of the prevalence, diversity, and catabolic versatility of soil bacteria, the information for HS bacterial degradation, such as degradative genes, catalytic enzymes, and metabolic pathways, is still insufficient.

In August 2011, many bacterial strains were isolated from the tundra grasslands in Nome, AK, owing to their ability to degrade humic acids. It was shown by 16S rRNA gene analysis that the strains are composed mainly of *Paenibacillus* spp. (79.5%) and *Pseudomonas* spp. (13.9%). Among them, strain PAMC 26794, with a high degradability for humic acids, was selected as a representative of this bacterial community. Based on the results of 16S rRNA gene similarity and comparative average nucleotide identity (ANI) analyses, PAMC 26794 was concluded to be phylogenetically closely related to *Paenibacillus* spp. The *Paenibacillus* sp. PAMC 26794 is believed to play a crucial function in *in situ* HS degradation and soil organic carbon cycling in the cold environment.

The genome of PAMC 26794 was analyzed using a 300-bp paired-end library (36,910,100 reads) with the Illumina HiSeq 2000 (Illumina, San Diego, CA), and a 7-kb paired-end library (189,549 reads) with the 454 GS FLX Titanium system (Roche Diagnostics, Branford, CT). The reads were assembled into 92 contigs using Genome Sequencer (GS) assembler 2.6 (Roche Diagnostic) and CLC Genomics workbench 5.0 (CLCbio, Denmark). The draft genome sequence of PAMC 26794 was approximately 6.7 Mb long with a G+C content of 46.03%. The resulting N_50_ size of the contigs was 164,684 bp, and the total coverage over the genome was 564-fold. Gene prediction and annotation using the Rapid Annotation using Subsystems Technology (RAST) pipeline (5) and Clusters of Orthologous Groups (COG) database (6) revealed 6,001 open reading frames (ORFs), 68 tRNA-encoding genes, and 5 rRNA genes in the draft genome.

### Nucleotide sequence accession numbers.

This Whole Genome Shotgun project has been deposited at DDBJ/EMBL/GenBank under the accession no. ANHX00000000. The version described in this article is the first version, ANHX01000000.
